# Positions in Parturition

**Published:** 1860-08

**Authors:** B. Dowler


### POSITIONS IN PARTURITION.

BY B. DOWLER, M. D.

The postures of parturient patients, and the attitudes of their assistants, as practiced a quarter of a century ago, and perhaps still in North-western Virginia, have not, so far as I know, been made public. These consist, as the sequel will show, chiefly of two groups, as witnessed during the last stage of labor—a stage easily recognized, not only by a digital examination, but by the cries or language of the patient, by her deep inspirations, with the holding of the breath, or a long breath, to give greater force to the extrusive actions of the expiratory and abdominal muscles; congestive flushings of the face, distention of the frontal, facial, and jugular veins; general muscular tention; the holding to and pulling by any thing within grasping distance during the expulsive pains, etc. These and other kindred phenomena, precursers of the impending crisis, show that the mental and bodily strain of child-birth has arrived; the sympathies of friends and attendants are excited. All, not excepting the accoucher, wish that this stage of the labor (agony it might be called) should be completed with the utmost speed consistent with the well-being of mother and child. The fœtal head no longer recedes, but produces more or less pressure, even in the intervals of the paroxysmal throes, the unusual prolongation of which give the medical attendant a degree of uneasiness which the preceding stage, however protracted, seldom if ever excites. Hence, every facility, in anywise calculated to aid the natural forces of the patient, is put in requisition. Even the appearance of physical help, though it may be intrinsically powerless, will, if harmless in itself, be highly beneficial, from its psychial influence in inspiring courage, confidence, and hope. With this view, the following plan was often adopted, particularly in rural practice, in the country above mentioned, and it probably still prevails.

The husband is now called in, sits on a chair, his wife on his lap; his knees separated, sometimes bound with a cloth, so that this separation may be the less fatiguing; two chairs are placed before the patient, some distance apart; she places her feet, having shoes on, so as to rest upon the rounds or frames of the chairs; two female assistants sit on these chairs, causing them to be immovable during the expulsive pains, while the helpers press with one hand, or with chest, against the patient's knees; with the other hand each takes one of the patient's hands, who pulls during the pains, and also presses her feet against the chairs, and her back against her husband, her knees being supported by her assistants, the accoucher sitting before the patient and between the assistants — a most convenient attitude, should his services be required.

During the expulsive throes, and indeed, all the time of the labor, the patient takes neither the horizontal nor vertical sitting posture, but is reclined or semi-recumbent, except when, during the intervals of the pains, she rises or changes her attitude to prevent fatigue.

This plan (which owes its origin, so far as I know, to the people) appears to give the woman a complete control over all the voluntary forces auxiliary to that of the uterus itself. But it fatigues all her attendants, except the doctor, and if the labor be not rapid, the husband is the greatest sufferer, next to herself, sometimes turning pale and faint, unless relieved by some of the attendants, who are always ready to aid in such emergencies. When the child is born, the patient is carried to bed, even though the placenta may not be delivered.

Another procedure is often adopted, by which the labor is conducted in bed. A bed is doubled up against inverted chairs, the bed being placed behind and next to the patient, a mattress being under her, upon which she rests in the half recumbent sitting posture, her feet resting against the foot-board; two sheets or towels are fastened to the two bed-posts at the foot of the bed, for her to pull by during the pains. Here, however, the foot-board is a barrier in the way of the accoucher, and to obviate this, the bed or other supports are placed against the wall, the patient lies semi-inclined across the matress, her limbs flexed, her feet resting on two chairs at the bed-side; upon the latter two assistants sit, to aid, as in the former position, the chair of the accoucher being placed between the latter, but a little in advance.

With proper arrangements, which need not be detailed further, in either of these positions the inferior limbs are flexed, the perineum and outlet unimpeded just as much as in the horizontal attitude, whether resting on the back or side, while, in the former, to use a common sense phrase of experienced matrons, the woman "helps herself" by the abdominal and expiratory muscular power to a greater extent than by simply lying down on her back. There is no more danger in the one case than in the other. In all the positions, good, bad, or indifferent, labor will "pursue the even tenor of its way" to a successful termination, with rare exceptions.

In the middle period of labor, the patient squeezes—in the last stage, pulls whatsoever she can take hold of—a mechanical advantage which she instinctively seeks, and which a physiologist of a different gender has no right to ignore or condemn as altogether superfluous. The obvious, materialistic muscular actions, both voluntary and involuntary, of parturition, afford the physiological obstetrician data illustrative of that vital and mechanical process infinitely more intelligible than the fanciful expositions of the so-called reflex actions of the "true spinal cord," or even the false.*

From a common sense point of view in the lying-in chamber, the "parturient acts," whether voluntary or involuntary, seem *direct muscular acts*, which take place about nine months after

* "The act of parturition never had been, and never could be, studied properly until the discovery of the physiology of the spinal marrow, by Dr. Marshall Hall. All the uterine motor actions are *reflex*," etc.—*W. Tyler Smith, M. D.*

conception, by primary or inherent laws of the living economy, which neither the parturient subject nor her accoucher can fathom, being ultimate, fundamental, self-evident, and not explicable by obscure language.

Neither danger to the child nor to the mother is likely to occur, whenever labor can be expedited by the natural forces of the mother; while, on the other hand, ergot, manipulations, and instrumental applications are more or less perilous to both, and, perhaps, in rare cases, the same may be said of even natural labor, when the head remains for many hours fixed or impacted in the pelvic bones, although, probably, the danger, if any, cannot be so great as is generally apprehended.

May I not here add a few words on this subject not originally intended in commencing this article? Although isolated individual experience may generally be of little import in opposition to that of the many, yet, judging from my own observations, I must say that, in cases wherein the presentation of the fœtal head is normal, or rather in that which is usually considered the most favorable position of the vertex, no danger to mother or child has resulted from severe and prolonged labor in which the child's head has rested apparently fixed low down, just within the perineum, and ready to make its exit—I say apparently fixed, because in the intervals of the pains the pressure of the head must necessarily intermit, though the head may not sensibly recede. The anatomical conformation of the mother, the position of the child, and the force of the pains being faultless, can anything be more trying to human patience and the moral sympathies, than a continuous series of pangs and disappointments, which, in rare cases, last perhaps an entire day! The accoucheur, relying upon nature, abstaining from all active or instrumental interferences, regulating the hegienic conditions of the chamber, attending to the conditions of the bladder, etc., inspiring hope, and sacrificing his valuable time, may suffer reproach, his skill being distrusted; but when a living child is born and the patient has a quick recovery, he will have an approving conscience, and may have an undamaged reputation among the judicious and right-thinking witnesses of his professional conduct. But as the world goes, glittering forceps, bloody craniotomies, and violent, quick deliveries, will usually pass for skill and superior qualifications. For the most part, the greatest use that an obstetrician can be put to, is to do nothing himself, and to prevent others from doing anything, mere placebos and "pious frauds" excepted.

Early in my professional life I had opportunities of witnessing or learning the natural history of prolonged labor, in which no active interference had taken place, in remote settlements in Harrison, Tyler and Lewis counties, Virginia, at distances sometimes more than forty miles from my residence, in Clarksburg. In some cases active labor had existed several days before my visits. In one of my first cases, I witnessed with dismay the active hard labor of a woman apparently in the last stage, the head of the child being fixed or locked during twenty-four hours after my arrival; fortunately, I had no instruments with me. The child was born alive, and the woman was wholly uninjured. I might detail other still more remarkable examples, in none of which accidents occurred. On the other hand, the unwarrantable interferences of some bold and ignorant midwives, and others, under apparently such circumstances, have resulted most deplorably, as I can testify.—*N. O. Medical and Surgical Journal.*

